# Effect of multiple allelic combinations of genes on regulating grain size in rice

**DOI:** 10.1371/journal.pone.0190684

**Published:** 2018-01-05

**Authors:** Umakanta Ngangkham, Sanghamitra Samantaray, Manoj Kumar Yadav, Awadhesh Kumar, Parameswaran Chidambaranathan, Jawahar Lal Katara

**Affiliations:** ICAR-National Rice Research Institute, Odisha, India; National Institute for Plant Genome Research, INDIA

## Abstract

The grain size is one of the complex trait of rice yield controlled by a plethora of interaction of several genes in different pathways. The present study was undertaken to investigate the influence of seven known grain size regulating genes: *DEP1*, *GS7*, *GS3*, *GW8*, *GL7*, *GS5* and *GW2*. A wide phenotypic variation for grain length, grain width and grain length-width ratio were observed in 89 germplasm. The correlation analysis showed a strong association among these three grain traits viz. GL, GW, GLWR and TGW which play important roles in determining the final rice grain size. Except for *GW2*, all six genes showed strong association with grain size traits. A total of 21 alleles were identified with an average of 2.1 allele/locus in 89 germplasm of which seven alleles were found to be favourable alleles for improving the grain size with the frequency range of 24 (26.97%) to 82 (92.13%); the largest was found in *GS5* followed by *GW8*, *GL7*, *DEP1*, *GS3* and *GS7* genes. Through ANOVA, four markers (GS3-*Pst*I, S9, GID76 and GID711) of three genes (*GS3*, *DEP1* and *GL7*) were found significantly associated with all the three traits (GL, GLWR and TGW). Concurrent results of significant associations of grain size traits with other markers were observed in both analysis of variance and genetic association through the general linear model. Besides, the population structure analysis, cluster analysis and PCoA divided the entire germplasm into three sub-groups with the clear-cut demarcation of long and medium grain types. The present results would help in formulating strategies by selecting suitable candidate markers/genes for obtaining preferred grain shape/size and improving grain yield through marker-assisted breeding.

## Introduction

Rice is one of the most important cereal crops of the world nourishing more than 50% of the world population [1]. It is also an economically important crop that accounts for ~20% of the world’s caloric intake [2]. Improvement of rice yield is one of the utmost necessities because of increase world population growth and availability of limited arable land worldwide. Rice yield, a complex trait is governed by numerous major and minor factors such as the number of productive tiller, panicle length, number of panicle per plant, grain weight, grain filling rate and grain size. Among them, the grain size is influenced by grain length, width and thickness which in combinations affect the grain weight. The physical appearance of rice grain which is one of the main components of rice grain quality is the major factors defining market values of the crop. Also, consumer preferences are mainly determined by the grain shape which in turn is the combination of traits such as grain length, width, thickness and the length-width ratio. Almost 80% of the world’s foods are derived from seeds of the staple crops including rice, wheat and maize [3] and it is worth to explore genes that function to define the seed traits including quality [4]. The knowledge available about the mechanisms of different partitioning of seed reserves into the major storage component is meagre [5].

The complete whole genome sequence of rice has largely facilitated in identification of several QTLs in rice. To date, over 8500 QTLs governing different agronomically important traits of rice including grain size have been mapped using various segregating populations generated from diverse parents [6]. In recent years, several major QTLs affecting the rice grain yield and size have been cloned and characterized. Some of the major genes/QTLs influence the rice yield include *GS3* [7,8], *GW2* [9], *qSW5/GW5* [10,11], *GS5* [12], *qGL3/qGL3*.*1* [13,14], *GW8* [15], *TGW6* [16] and *GL7/GW7* [17,18], *Gn1a* [19], *DEP1* [20], *OsSPL14* [21,22], etc. Among them, the major QTL, *GS3* for grain length encodes a protein containing putative the PEPB-like domain, a transmembrane region, a putative TNFR/NGFR family cysteine-rich domain and a VWFC module. A plant-specific organ size regulation (OSR) domain in the N-terminus of *GS3* is both necessary and sufficient for functioning as a negative regulator of grain length and a point mutation in the second exon leads to a 178 amino acids truncation in the C-terminus of the predicted protein thereby increasing the grain length and weight [8]. For grain width, *GW2* was identified [9] which encodes a RING-type E3 ubiquitin ligase and loss of function leads to increase in grain width and weight. This gene also acts as a negative regulator for grain width through the control of cell division in the spikelet hull by targeting unknown substrate for the ubiquitin-dependent degradation by the 26S proteasome. Fine mapping of this locus detected a 1212-bp deletion associated with the increased grain width [11]. Another important QTL for grain width, *GW5* encodes a novel nuclear protein of 144 amino acids that is localized to the nucleus and likely acts in the ubiquitin-proteasome pathway to regulate cell division during seed development with a 1212-bp deletion associated with the increased grain width [11]. Among the other cloned genes for grain size, q*SW5* [10], *qGL3*.*1* [13], *GIF1* [23] and *TGW6* [16], *GS5* [12] and *GW8* [15] were reported as major regulator for grain size. From the recently cloned and characterized genes, it is revealed that multiple signalling pathways such as ubiquitination-mediated proteasomal degradation, phytohormones, and G-protein signalling pathways are involved in the determination of grain length [24]. However, the molecular mechanism underlying the controlling of grain size are still unclear.

The present research was undertaken to identify marker–trait association for grain size traits in rice. It was hypothesized that diverse rice germplasm varied widely in grain size due to presence of different combination of multiple alleles of several genes/QTL related to grain size and these candidate genes were significantly associated with the trait of interest in rice. In the present study, we evaluated 89 rice germplasm with diverse grain length and width to find out the allelic variation of seven major grain size regulating genes for grain size through candidate gene-based association approach. Further, relatedness among rice germplasm based on gene-based molecular markers was investigated to dissect the genetic architecture and heritability of grain size.

## Materials and methods

### Plant materials and phenotyping

A set of 89 germplasm showing different grain shape and size were used in this experiment. The seeds were sown in the nursery and transplanted in the field with the normal spacing of 10×15 cm with the recommended agronomic practices. At the stage of plant maturity, grain length (GL) and grain width (GW) were recorded (in mm) as average from 5 completely filled and matured grain samples by using digital vernier caliper. The grain length-width ratio (GLWR) was estimated by dividing mean grain length with mean grain width of each germplasm. The 1000 grain weight (TGW) was measured using electronic digital weighing balance by taking 100 filled grains from main panicle of each sample and the value were multiplied by 10 factors to get 1000 grains weight. The frequency distribution for GL, GW, GLWR and TGW, and their linear correlation coefficient (r) were analysed using statistical package XLSTAT (https://www.xlstat.com).

### Genomic DNA isolation

DNA was isolated from young leaf tissues using the cetyltrimethyl ammonium bromide (CTAB) method. The purified genomic DNA was checked for quantity and quality on 0.8% agarose gels electrophoresis and NanoDrop ND-1000 Spectrophotometer (Thermofisher scientific, USA). The plant DNA samples were diluted with nuclease-free water to the working concentration of 20 ng/μl for PCR amplification.

### Genotyping for grain size related genes

The rice germplasm were mined for the presence of 7 grain size related genes using 10 functional/linked markers. The grain size related genes included in the present study are *DEP1*, *GS7*, *GS3*, *GW8*, *GL7*, *GS5* and *GW2*. The detailed primer pairs’ information of the eleven markers used in this study is given in Table 1. PCR reaction was carried out in a 25μl of a solution containing 1X T*aq* buffer (10 mM Tris-HCl, 50 mM KCl, pH 8.3), 0.2 μM of each forward and reverse primers, 1.5 mM MgCl_2,_ 0.2 μM of each of dNTP, 20 ng template DNA, and 1U of *Taq* DNA polymerase (Dream T*aq*, Thermo Scientific, USA). The PCR condition was set up as follows: 94°C of 5 min for initial denaturation followed by 35 cycles of 94°C for 45 sec, primers annealing for 30 sec at varied temperatures and elongation for 1 min at 72°C, followed by a final elongation at 72°C for 10 min. The PCR products were analyzed by electrophoresis in 1.5% agarose or 3.5% Metaphor agarose gels (Lonza, USA) stained with ethidium bromide together with a 100 bp DNA ladder. After electrophoresis, the samples were documented using a gel documentation system (AlphaImager, USA). All marker types were visually scored for their presence (1) or absence (0). All PCR reactions were repeated at least twice to cross-check the scoring data.

**Table 1:** Details of the 10 molecular markers for 7 grain size regulating genes used in the present studied.

S. N.	Genes	Markers	Forward (5'to 3')	Reverse (5'to 3')	Tm (^o^C)	Types	References
1	*DEP1*	S9	TGGACACTTGTTATCTTCTCAT	AACTGGAAGTTTGTAACACTCA	60	STS	Huang et al 2009
2	*GS7*	FMGS7	TGGTCAAATCATGGGCTAAT	TATTATTGTGCCTGCGATCC	55	STS	Shao et al 2012
3	*GS3*	GS3-P*st*I	TATTTATTGGCTTGATTTCCTGTG	GCTGGTTTTTTACTTTCATTTGCC	60	CAPS	Yan et al. 2011
4		RGS1	TCCACCTGCAGATTTCTTCC	GCTGGTCTTGCACATCTCTCT	55	SSR	Wang et al 2011
5		SR17	TGCCCATCTCCCTCGTTTAC	TGTTCGTTGCTGGTGTTG	55	InDel	Wang et al 2011
6	*GW8*	GW8-InDel	TTGTGATGGCAATTAGTAAGCAG	GTTCTCCAGCTCGTCGGCTA	57	InDel	Wang et al 2012
7	*GL7*	RID76	CACCGAAGACTGATCAGCAA	TCACATTCGAGTGGAGCAAC	58	InDel	Bai et al 2010
8		RID711	GCACATGCATGCTAGGACAT	AGCCGGTAAATTTCTTGCAC	58	InDel	Bai et al 2010
9	*GS5*	RM574	AAACTAGCCACGGTTTGGTAGGG	AGGGTGGCAGGGATGTAATTTCC	55	SSR	Li et al 2011
10	*GW2*	W004	ACCAGCATTCAGCCATTC	TTGATAGAGCAACCCAGT	60	STS	Song et al 2007

### CAPS markers (GS3-*Pst*I) of *GS3* gene analysis

The PCR amplification was carried out using GS3-*Pst*I primer as explained above. The PCR products were cleaved with *Pst*I restriction enzymes (New England Biolabs, Inc., USA), following the manufacturer’s protocol. The digestion mixture was set for 10 μl by adding 5μl of PCR product, 1μl of restriction enzyme (3U), and 1μl of 10 X buffer with additional nuclease free water to make the final volume to 10μl. The reaction mixture was incubated at specific temperature based on enzyme’s requirement for 2 hours. The digested product was separated in 1.5% agarose gel, observed in UV Transilluminator and bands were scored similar to SSR marker scoring.

### Allele scoring and diversity analysis

The eleven markers were scored as present (1) or absent (0) to generate a binary matrix for each individual and used to infer the assessment of genetic distance and similarity coefficients. An unweighted neighbour joining un-rooted tree was constructed using computer software programme Darwin [25]. The Polymorphism information content (PIC) refers to the relative value of each marker regarding the amount of polymorphism exhibited. The estimation of major allele frequency, allele per locus, gene diversity, heterozygosity, PIC value of the markers was determined using PIC calculator (http://www.liv.ac.uk/~kempsj/pic.html); [26] and POWERMARKER Ver3.25 program [27].

### Statistical analysis

The association between grain size (GL, GW, GLWR and TGW) and 10 selected markers of 7 genes were analyzed using the general linear model (GLM) model in TASSEL5 software [28]. To study the presence of genetic structure for grain size, population structure analysis was performed using the program STRUCTURE version 2.3.4 [29]. The model was run based on an admixture model with correlated allele frequencies and the number of sub groups (K) in the clusters was determined by simulating different K-values (K = 1 to 10) with 5 independent runs and run length of 100,000 burn-in period and 100,000 MCMC. The optimal K-value was determined through the ΔK method [30] using Structure Harvester ver. 0.6.193 application [31]. The Principal Coordinate Analysis (PCoA) was calculated from the generated binary data of markers with the GenAlEx 6.502 [32]. The genotypic data of genes corresponding to the grain size were converted into binary matrix which was used to determine the analysis of molecular variance (AMOVA) for separation of the total molecular variance between and within groups and significant F_ST_ using GenAlEx 6.502 [32]. All other statistical analysis was conducted using statistical package XLSTAT (https://www.xlstat.com).

## Results and discussion

The rice seed is a complex storage structure containing different types of tissues. Determination of seed size involves control of growth of the embryo, the surrounding triploid endosperm and seed coat. The yield and nutritional value of rice is mostly determined by the synthesis and storage of carbohydrates, proteins and minerals during grain filling and culinary quality is affected by the interaction of various enzymes to produce the final structure of the starch at molecular and granule level [33]. Consequently, it is difficult to achieve large increases in seed yield by altering single or only a few genes. Though a number of QTL/genes were cloned and characterized for grain size, the effects of different allelic combinations from different genes to determine the final grain shape and size are still unclear.

### Phenotypic variations and correlation coefficient analysis for the grain size traits

The phenotypic variation in grain size was determined in 89 rice germplasm. A substantial variation in grain size of 89 germplasm suggests a quantitative inheritance governed by multiple genes which indicated the population panel of 89 germplasm was sufficient enough to undertake further association studies. The phenotypic data for grain length, grain width, length-width ratio and 1000-grain weight are given in S1 Table. Estimates for range, mean, standard deviation and coefficient of variation (CV %) for 89 germplasm evaluated in the present study are depicted in Table 2. The mean GL of the 89 germplasm was found to be 8.19± 2.16 mm. The 89 germplasm showing an appreciable range for GL to the tune of 7.71 mm (5.070 to 12.78 mm) suggest that apart from one or two major genes there might be some interactions between the additional genes present in the germplasm and the environment. Similarly, the mean grain width of the 89 germplasm were recorded to be 2.399±0.352 mm. Compared to GL, grain width had a lower range of variation of 1.64 as expected and extended from 1.71 to 3.35 mm. Likewise, in case of GLWR, the mean was found to be 3.50 with a range from 1.77 to 5.55. The mean TGW of the 89 germplasm was found to be 21.54± 5.516 g with a wide range of variation of 21.1. The Coefficient of variations (CV %) of GL, GW, GLWR and TGW showed 26.42%, 14.67%, 31.39% respectively. The GL, TGW and GLWR showed the largest phenotypic variation as compared to the GW. Since the availability of genetic variability for yield related components in the germplasm could be a valuable selection priorities of breeders for need based breeding in rice yield improvement, it is possible to effectively utilize the studied germplasm of the varied grain length and grain length-width ratio effectively in rice yield enhancement.

**Table 2:** Descriptive statistics for grain size traits in 89 rice germplasm.

Traits	range	Mean	Std. deviation	Coefficient of variation (%)
GL	7.71	8.193	2.165	26.42
GW	1.64	2.399	0.352	14.67
GLWR	3.78	3.502	1.099	31.39
TGW	21.1	21.54	5.516	25.60

The Pearson correlation coefficients (r) were calculated for pairwise analysis among the GL, GW, GLWR and TGW traits (Table 3) using 89 diverse germplasm. The analyses of correlation between the GL and GW showed a negative correlation between them which is also found weak showing r = -0.164 at *P* value <0.1. Such linear negative correlation had also been reported in other grain yield or yield related studies [34,35,36]. However, the analyses of correlation of GLWR with GL, GW and TGW showed strong and highly significant correlation among them. A highly significant and strong positive correlation between GL and GLWR (r = 0.908 at *P* value <0.001), GL and TGW (r = 0.577 at *P* value <0.001) indicates the GLWR and TGW are 90.8% and 57.7% respectively positively influenced by the grain length. Similarly, a strong negative significant correlation between GW and GLWR (r = -0.543 at *P* value <0.001) showed a negative reduction in grain width when GLWR is 54.3%. This result suggests that there is a strong association among the four grain traits which play important roles in determining the final rice grain size. Such patterns are in accordance with the concept that GLWR contributes the major effects in combining the length and width of the rice grain. Further, the whole 89 germplasm were divided into three groups based on grain length: short grain with <6 mm long (14), medium grain with 6–9 mm long (35) and long grain with >9 mm long (40). It was observed that the highest number of germplasm were found in the group of long grain type followed by medium and short grain types. The average length of short, medium and long grain types were found to be 5.56 mm, 6.65 mm and 10.46mm, respectively. However, the studied germplasm possessed the minimum grain length of 5.07 mm and a maximum length of 12.78 mm.

**Table 3:** Correlation coefficients analysis among the four traits for grain size.

Traits	Grain length (L)	Grain width (W)	GLWR
**Grain length**	1	-	-
**Grain width**	-0.164*	1	-
**GLWR**	0.908**	-0.543**	1
**TGW**	0.577**	0.103	0.424**

**Correlation is significant at the *P* value <0.001

*Correlation is significant at the *P* value <0.1

### Allelic contribution of seven grain size regulating genes for grain size

**Fig 1:**
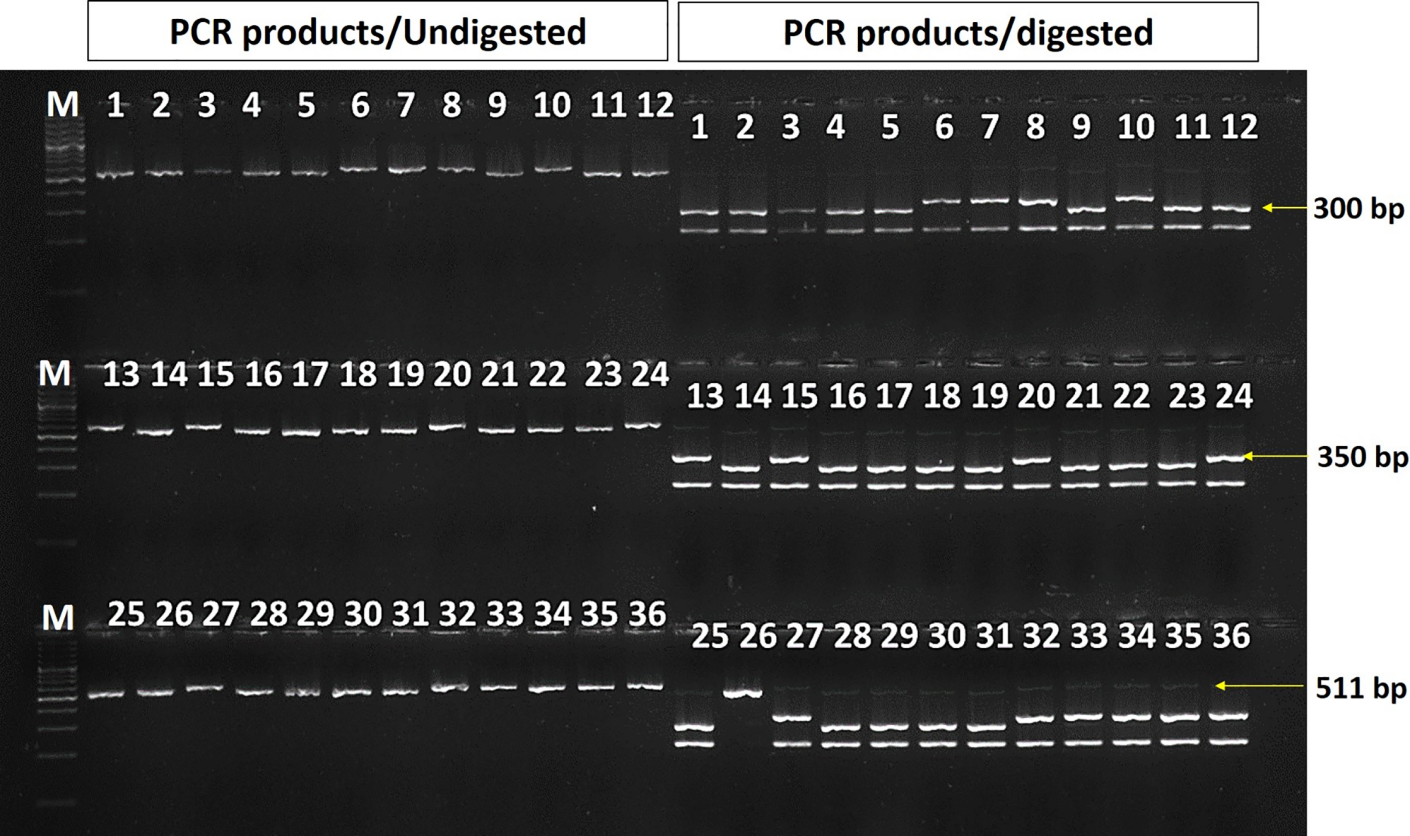
Profiling of GS3-*Pst*I functional CAPS marker in representative rice germplasm. The digested PCR fragments were separated in 1.2% agarose gel. Lane: M—100 bp DNA ladder, 1–36 represents rice germplasm. The size of the DNA fragments is indicated on the side of the figure.

**Fig 2:**
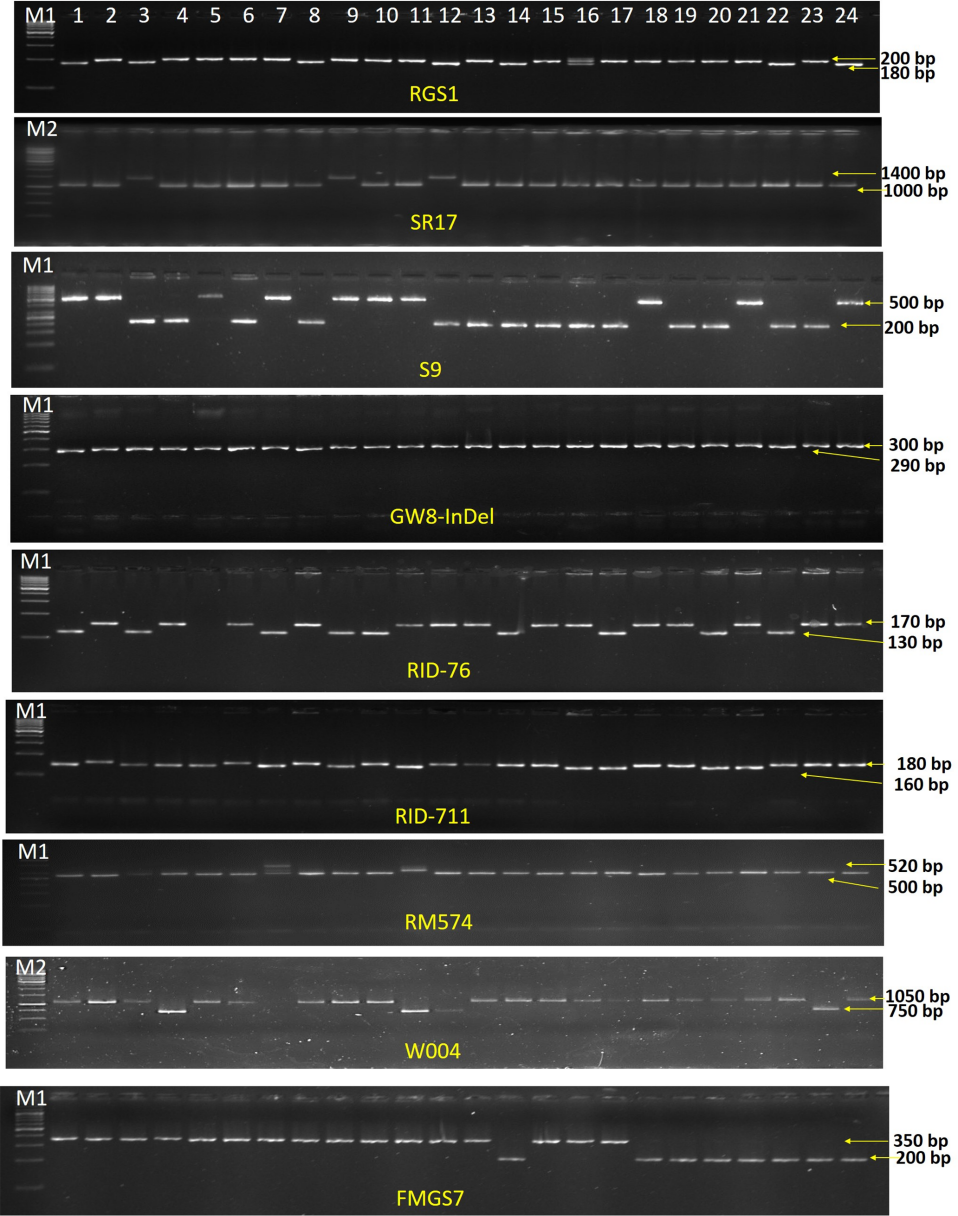
Representatives of PCR amplified fragments of linked/functional markers of 7 genes. Lane: M1–100 bp DNA ladder, M2 – 1Kb DNA ladder, 1–24 represents rice germplasm. The size of the DNA fragments is indicated on the right side of the figure.

Understanding the allelic diversity of grain size regulating genes along with their relationships and phenotypic effects would be highly useful for genetic manipulation in increase of grain size in rice. The allelic distribution of seven genes related to grain size regulating traits was examined in 89 diverse rice germplasm using ten genic /linked markers. Among them, the *GS3* gene localized on chromosome 3 has been reported as the most important gene for grain size (grain length) regardless of genetic background [37] which encodes a protein with a putative phosphatidylethanolamine-binding protein (PEBP)-like domain, a transmembrane domain, a putative tumor necrosis factor receptor (TNFR)/nerve growth factor receptor (NGFR) family domain and a von Willebrand factor type C (VWFC) module [38]. In this study, three genic functional markers (GS3-*Pst*I, RGS1 and SR17) were used to assess the allelic pattern in *GS3* locus in 89 germplasm (Table 4). The GS3-*Pst*I is a functional CAPS markers using *Pst*I endonuclease enzyme differentiating ‘C/A’ SNP mutation in second exon of *GS3* gene which produces a truncated protein without functional domain that associated with an enhanced rice grain length [39,40,41]. Using GS3-*Pst*I primers, all 89 germplasm generated PCR products of approximately 511 bp in size (Fig 1). Since CTG**C**AG sequence (restriction site of *Pst*I enzyme) is present in the PCR products, the 511 bp PCR products was digested by *Pst*I endonuclease enzyme into 300 bp smaller fragments, thereafter referred as C-allele. Some of the germplasm produced additional allele with digested PCR fragment of 350 bp which was referred here as B-allele. Similar type of additional allele of *GS3* gene was also observed in rice germplasm which might be due to insertion of 45-base pair in the first intron of *GS3* gene [42]. Besides, the presence of CTG**A**AG sequence in the PCR products which couldn’t be digested by *Pst*I restriction enzyme, was referred as A-allele. Among 89 germplasm used, 33, 32 and 24 germplasm were found to possess A-, B- and C-alleles, respectively which also exhibit significant differential grain size. The highest frequency of 37.07% was observed for A-allele followed by B- (35.95%) and C- (26.96%) alleles. The germplasm possessing A-allele also has higher mean grain length (10.18±1.49 mm), mean grain length-width ratio (4.42±0.89) and 1000-grain weight (24.45±4.12 g) as compared to other two alleles (Table 4). Interestingly, the B-allele with 45 bp insertion in the intron was found to be intermediate GL, GLWR and TGW compared to the germplasms carrying the other two alleles. The germplasm carrying B-allele had medium grain length (7.58±1.71 mm) significantly longer mean GL (17.15%), higher mean GLWR (17.75%) and higher 1000-grain weight (12.78%) as compared to the C-allele. This B-allele would be a valuable allele for efficient selection of medium grain length type which possess a desirable processing trait for high yield of milled rice grains as phenotypic selection is really a difficult process [43]. It was explained that an aberrant splicing in the intronic regions in the B-allele led to a translation of novel modified GS3 protein with different functional domains determining the grain length in rice [42,44,45]. Moreover, these three alleles of GS3-*Pst*I marker of *GS3* gene were found to be significantly associated with differences in grain length, grain length/width ratio and 1000-grain weight traits except grain width trait in 89 rice germplasm. Comparison of this allelic series in the *GS3* gene provides the best information about the relationship of extent of quantitative variation in grain size regarding with the changes in the gene at different sites. The RGS1 is a SSR marker with (AT)n motif in the last intron of *GS3* gene produced 196 (13 AT repeats) and 180 bp (5 AT repeats) fragments which corresponded to A and B alleles, respectively [43]; these two alleles of RGS1 markers were detected in 89 rice germplasm with frequency of 65.16% (A-allele) and 34.84% (B-allele) (Fig 2). A-allele occurs at a high frequency, nearly twice that of B-allele. Notably, this marker was not found to be associated in any trait components of the grain size in the present study (Table 4). The SR17 marker was developed based on an insertion/deletion of 338 bp in the second intron of *GS3* gene [43]. This marker produced two different PCR products of 1.4 kb and 1.1 kb fragment size in 89 germplasm which corresponded to two alleles A and B respectively. The B-allele was observed in high frequency of 85.39% in all germplasm while A-allele with 14.61% was found only in 13 rice germplasm used in this study. Similarly, distorted frequency of alleles of SR17 markers of *GS3* gene were also observed in 287 Chinese rice accessions [43]. Similar to the RGS1 marker, SR17 marker also showed no association with any trait components of the grain size in the present study (Fig 2, Table 4). Among the three markers of *GS3* gene, only the allelic variations of GS3-*Pst*I markers were found to be highly associated with grain length, grain length-width ratio and 1000-grain weight, consistent with the previous reports [37,42,43] and hence, would be very valuable for analysing functional diversity and improvement of rice yield by breeding through marker-assisted selection.

**Table 4:** Analysis of allelic variation of 7 grain regulating genes in 89 germplasm.

					Grain length (mm)		Grain width (mm)		Grain Length-Width ratio		1000-Grain Weight (g)	
S No.	Genes	Markers	Alleles	germplasms	Means±SD	*P* value	Means±SD	*P* value	Means±SD	*P* value	Means±SD	*P* value
1	*GS3*	GS3-*Pst*I	A	33	10.18±1.49^a^	<0.0001*	2.35±0.27 ^a^	0.591	4.42±0.89 ^a^	<0.0001*	24.45±4.12^a^	<0.0001**
			B	32	7.58±1.71^b^		2.42±0.37 ^a^		3.21±0.91^b^		20.97±4.92^b^	
			C	24	6.28±0.95^c^		2.44±0.42 ^a^		2.64±0.54^c^		18.29±6.02^c^	
		RGS1	A	58	8.34±2.33^a^	0.394	2.38±0.35^a^	0.388	3.59±1.15^a^	0.289	21.36±6.02^a^	0.681
			B	31	7.92±1.82^a^		2.44±0.36^a^		3.33±0.99^a^		21.87±4.48^a^	
		SR17	A	13	8.19±1.88^a^	0.994	2.39±0.36^a^	0.889	3.58±1.21^a^	0.794	22.31±4.35^a^	0.588
			B	76	8.19±2.22^a^		2.40±0.35^a^		3.49±1.09^a^		21.41±5.70^a^	
2	*DEP1*	S9	A	44	7.55±2.02^a^	0.005*	2.41±0.37^a^	0.676	3.21±1.06^b^	0.013*	19.93±5.83^a^	0.006**
			B	45	8.83±2.14^b^		2.38±0.33^a^		3.78±1.08^a^		23.11±4.74^b^	
3	*GW8*	GW8-Indel	A	22	6.99±1.72^b^	0.002*	2.45±0.43^a^	0.415	2.92±0.81^b^	0.004*	19.95±6.62^a^	**0.121**
			B	67	8.58±2.16^a^		2.38±0.32^a^		3.69±1.11^a^		22.06±5.04^a^	
4	*GL7*	GID76	A	65	8.71±2.17^a^	<0.0001*	2.39±0.33^a^	0.821	3.73±1.13^a^	0.001*	22.40±4.92^a^	0.014*
			B	24	6.78±1.40^b^		2.41±0.42^a^		2.87±0.70^b^		19.20±6.42^b^	
		GID711	A	62	8.63±2.06^a^	0.003*	2.39±0.33^a^	0.697	3.71±1.11^a^	0.007*	22.51±4.70^a^	0.011*
			B	27	7.19±2.09^b^		2.42±0.41^a^		3.03±0.94^b^		19.30±6.60^b^	
5	*GS5*	RM574	A	7	6.65±1.57^b^	0.049*	2.45±0.45^a^	0.667	2.83±1.02^a^	0.090	19.65±6.63^a^	0.349
			B	82	8.32±2.16^a^		2.39±0.35^a^		3.56±1.09^a^		21.70±5.42^a^	
6	*GW2*	W004	A	65	8.39±2.22^a^	0.163	2.37±0.34^a^	0.227	3.62±1.13^a^	0.089	21.44±5.51^a^	0.778
			B	24	7.66±1.94^a^		2.47±0.37^a^		3.18±0.97^a^		21.81±5.63^a^	
7	*GS7*	FMGS7	A	65	7.67±1.96^b^	<0.0001*	2.43±0.37^a^	0.151	3.24±1.03^b^	<0.0001*	20.75±5.72^b^	0.026*
			B	24	9.60±2.11^a^		2.31±0.27^a^		4.20±0.99^a^		23.67±4.32^a^	

*ANOVA test for significant at the level of *P* value <0.05.

^a, b^ and ^c^ were ranked by Tukey (HSD) test

The *DEP1* gene, encodes a previously unknown PEBP (phosphatidylethanolamine-binding protein) like domain protein sharing some homology with the N terminus of *GS3* gene and regulates panicle architecture for which a STS marker S9 was also developed [20]. Two alleles of *DEP1* genes, A and B were distinguished by two different PCR products of 500 and 200 bp fragment size, respectively which were detected in 89 rice germplasms with similar frequency of 49.43% (A-allele) and 50.54% (B-allele) as well (Fig 2). The B-allele had significantly longer mean grain length (8.83±2.14 mm), higher mean GLWR (3.78±1.08) and more mean TGW (23.11±4.74g) as compared to the A-allele **(**Table 4**)**. The allelic variation of *DEP1* gene showed significantly associated with the difference in grain length, grain length/width ratio and 1000-grain weight traits in 89 rice germplasm except grain width. Therefore, this marker could be effectively utilized for efficient selection of grain length and weight traits similar to previous described *GS3* gene.

The *GW8* gene synonymous with *OsSPL16*, encodes a protein of an SBP-domain transcription factor that regulates grain size by positively regulating the cell proliferation of grain [15]. The GW8-indel marker was developed based on 10-bp deletion in the promoter region which was reported to be responsible for grain size variation. Based on this 10-bp deletion, this markers generated two alleles, A and B corresponded to Basmati- and *indica*-type alleles, respectively [15,42]. The A- (Basmati) allele was found in 22 germplasm and B- (*indica*) allele in 67 germplasm **(**Fig 2, Table 4**)**. Remarkably, B-(*indica*-type) allele which had longer mean grain length (8.58±2.16 mm) and higher mean GLWR (3.69±1.11) as compared to the A-allele was corroborated with previous report [42]; significant differences between the alleles for GL and GLWR were observed.

The *GL7* gene also known as *GW7*, encodes a TONNEAU1-recruiting motif protein with similarity to C-terminal motifs of the human centrosomal protein CAP350 and regulates the grain shape by increasing cell division in the longitudinal direction [18]. Here, two linked InDel markers, GID76 and GID711 were used to determine the allele distribution of *GL7* gene in the 89 germplasm **(**Fig 2, Table 4**)**. The two alleles of GID76 were able to differentiate the whole germplasm into 65 (73.03%) and 24 (26.97%) with A- and B-alleles respectively. Similarly, GID711 markers produced maximum frequency (69.66%) of A-allele and minimum frequency (30.34%) of B-allele. The A-alleles of both the InDel markers had longer mean grain length, higher mean GLWR and mean TGW as compared to their corresponding B-alleles. These markers showed their significant association with grain length, grain length/width ratio and grain weight which could be used for efficient selection for GL, GLWR and TGW traits.

The *GS5* gene was fined map on chromosome 5 to 11.6-kb region between RM574 and S2 markers containing single ORF which encodes a putative serine carboxypeptidase and its higher expression functions as a positive regulator of grain size by regulating grain width, filling and weight [12]. Due to the unavailability of effective functional marker of *GS5* gene, we used the closest/linked marker, RM574 for assessing the germplasm. This marker yielded two alleles, A and B with the frequency of 7 (7.86%) and 82 (92.14%) respectively (Fig 2, Table 4). The germplasm carrying the B-allele had significantly longer mean grain length as compared to another one. This marker did not show any association with GW, GLWR and TGW. In contrast to our results, Lee et al [42] could achieve three type of alleles by employing the markers developed from the promoter region of *GS5* gene which showed significant association with all the grain size traits. This result indicates that the RM574 is not efficient enough to determine the allelic distribution of *GS5* gene for rice grain size in comparison to the genic variations in the promoter sequence [42,46].

The *GW2* gene, a major gene that controls rice grain width and weight encodes a previously unknown RING-type protein with E3 ubiquitin ligase activity, regulates grain size by increasing cell numbers, resulting in enhanced grain width, weight and yield [9]. A linked marker, W004 was used to detect the distribution of *GW2* in 89 germplasm and detected two alleles with frequency of 73.03% (A) and 29.94% (B) (Fig 2, Table 4). However, there was no significant difference observed for these alleles with the grain phenotypes indicating that this marker should not be used for which InDel [9] and SNPs [47] markers could be utilized for detecting the allelic variations of *GW2* gene in rice germplasm.

Another important QTL for grain size, *GS7* gene was identified on chromosome 7 that determines the extra extend of grain length with the combination of the favourable allele of *GS3*gene [48]. In our study, the functional InDel marker FMGS7 based on *GS7* gene distinguished 89 germplasms into two allele (A and B) groups with frequency of 73.03% and 26.97% respectively. The B-allele has significant higher mean grain length (9.60±2.11 mm) as compared to mean grain length (7.67±1.96 mm) of A-allele (Fig 2, Table 4). The B-allele has also higher mean TGW (23.67±4.32g) as compared to mean mean TGW (20.75±5.72g) of A-allele. Unlike to Shao et al [48], no association was observed with variation in grain width and the ratio of length to width. Shao et al [48] reported that germplasm carrying same A-allele of *GS3* gene along with different alleles of *GS7* gene combinations significantly produce different grain length in rice. In the present study, 33 germplasm out of 89 germplasms were found to carry A-allele of *GS3* gene. Using the FMGS7 marker, 33 genotypes again could be differentiated into two groups (GS3/GS7 alleles): group 1 as A/A alleles (19 germplasm) produced mean grain length of 9.86±1.13 mm and group 2 as A/B alleles (14 germplasm) produced mean grain length of 10.59±1.83 mm. However, there were no significant differences observed in grain length between these two groups. The discrepancy in the present result might be either due to uses of small size population or allelic variation of other unknown genes that controls the complex traits of grain size.

Thus, the six genes out of seven genes used in the present study showed strong association with grain size traits. The occurrence of allelic variation and association with one or more grain size traits such as GL, GW, GLWR and TGW would be useful to evaluate allele-specific markers for yield enhancing genes through MAS in rice breeding.

### Favourable alleles of seven grain regulating genes

**Fig 3:**
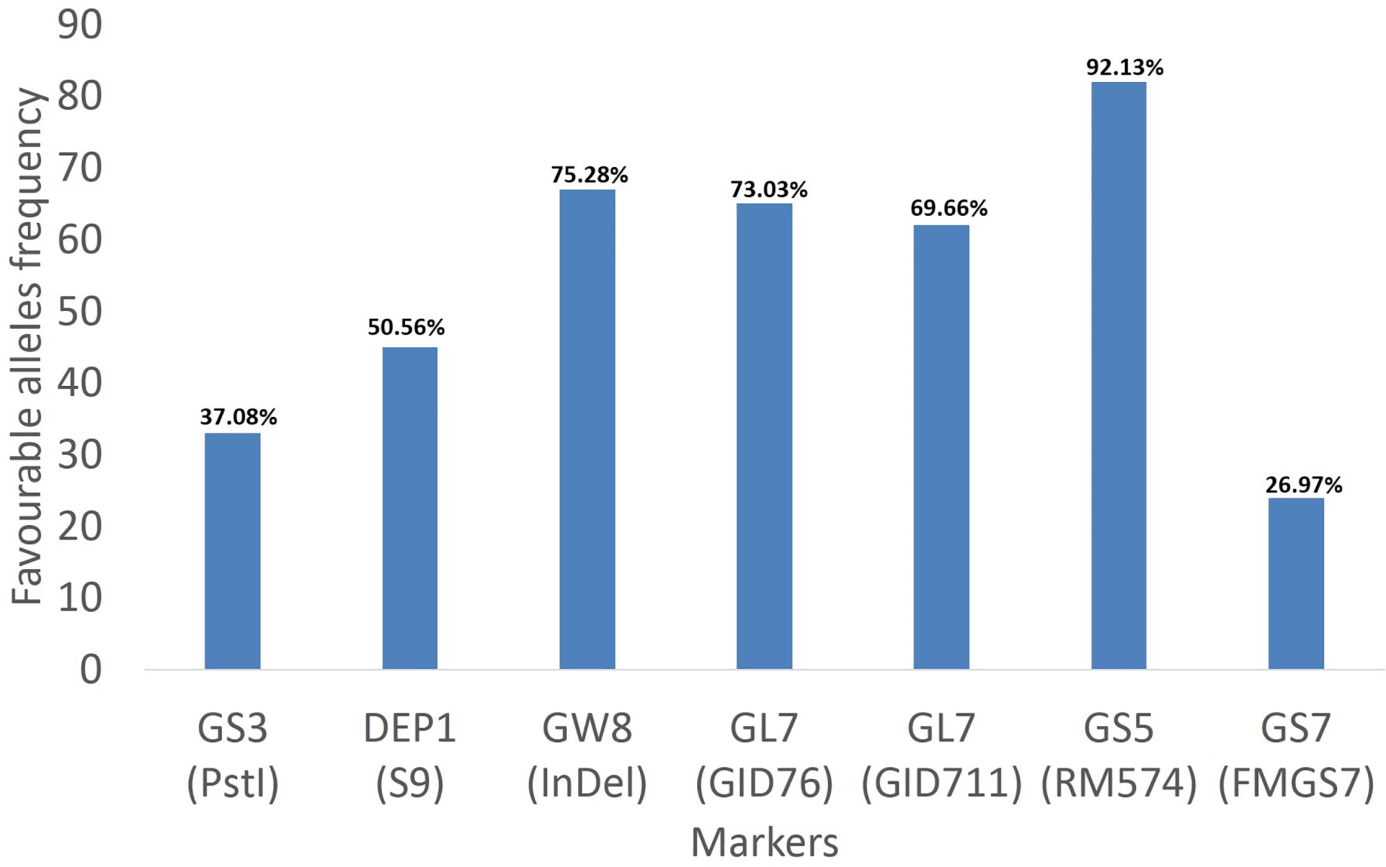
Frequency distribution of favourable alleles of six genes related to grain size in 89 rice germplasm.

Identification of favourable alleles of the trait of interest is one of the prerequisite to enhance the performance of modern cultivars by introgressing and cumulating several favourable alleles from the vast gene pool of rice germplasm in the breeding population of rice through molecular markers. These favourable alleles of grain size regulating genes would be useful for understanding the rice seed development and improving grain size, thereby increasing the rice yield through MAS approach. The individual contribution of these seven genes (*GS3*, *DEP1*, *GW8*, *GL7*, *GS5*, *GW2* and *GS7*) towards grain size were estimated and the cumulated contribution of their favourable alleles for grain size has been thoroughly understood. In the present study, a total of 21 alleles were identified at the 10 loci of seven genes with an average of 2/locus in 89 germplasm (Table 4). Of which, seven alleles were found to be beneficial/favourable alleles for improving the grain size through GL, GLWR and TGW. ANOVA was used to estimate the favourable allele for each locus by determining the significant difference of means between rice accessions with the favourable alleles (FAs) and non-favourable alleles (N-FAs) (Table 5). The frequency of favourable alleles were found to be in the range of 24 (26.97%) to 82 (92.13%) with the largest in *GS5* followed by *GW8*, *GL7*, *DEP1*, *GS3* and *GS7* genes (Fig 3). Six genes were observed to be significantly associated with the grain length. Seven markers of six genes (*GS3*, *DEP1*, *GW8*, *GL7*, *GS5* and *GS7*) showed the significant difference for GL and GLWR (*P*<0.05) and these seven alleles were appeared to be favourable alleles found in 89 rice germplasm (Table 5). A significant positive correlation exists among GL, GLWR and TGW and the same alleles were observed to be favourable for both the GL and GLWR traits. All the favourable alleles of six genes contributed significantly higher for GL and GLWR traits in 89 rice germplasm. Similarly, all the favourable alleles except for *GW8* and *GS5* genes showed similar positive contribution for TGW. Unlike to other FAs, the favourable allele of the only *GS5* gene do not contribute toward GLWR and TGW trait. The means that GL, GLWR and TGW values of cumulated FAs were found to be significantly higher as compared to cumulated N-FAs (Table 5) indicating that the pyramiding of favourable alleles leads to increase grain length and grain weight, and thereby improving the grain yield in rice. Similarly, pyramiding of favourable alleles has been reported to lead higher stem water-soluble carbohydrates and higher 1000-grainweight in rice [49]. Therefore, pyramiding of favourable alleles for obtaining genotypes with higher grain size through marker-assisted selection paves a way to overcome the yield bottleneck in rice breeding.

**Table 5:** Pyramiding of favourable alleles of six genes for GL and GLWR.

			Grain Length (mm)		Grain Length-width ratio		1000-Grain Weight (g)	
Genes	Markers	Alleles	FA (Means±SD)	N-FA (Means±SD)	FA (Means±SD)	N-FA (Means±SD)	FA (Means±SD)	N-FA (Means±SD)
*GS3*	GS3-*Pst*I	**A**	10.18±1.49^#^		4.42±0.89^#^		24.45±4.12	
		B	-	7.58±1.71	-	3.21±0.91		20.97±4.92
		C	-	6.28±0.95	-	2.64±0.54		18.29±6.02
*DEP1*	S9	A	-	7.55±2.02	-	3.21±1.06		19.93±5.83
		**B**	8.83±2.14^#^	-	3.78±1.08^#^	-	23.11±4.74	
*GW8*	GW8-Indel	A		6.99±1.72	-	2.92±0.81	-	-
		**B**	8.58±2.16^#^	-	3.69±1.11^#^	-	-	-
*GL7*	GID76	**A**	8.71±2.17^#^	-	3.73±1.13^#^	-	22.40±4.92	
		B	-	6.78±1.40	-	2.87±0.70		19.20±6.42
	GID711	**A**	8.63±2.06^#^	-	3.71±1.11^#^	-	22.51±4.70	
		B	-	7.19±2.09	-	3.03±0.94		19.30±6.60
*GS5*	RM574	A	-	6.65±1.57	-	-	-	-
		**B**	8.32±2.16^#^	-	-	-	-	-
*GS7*	FMGS7	A	-	7.67±1.96	-	3.24±1.03		20.75±5.72
		**B**	9.60±2.11^#^	-	4.20±0.99^#^	-	23.67±4.32	
**Means±SD**			**8.98±0.66**^**a**^	**7.08±0.50**^**b**^	**3.92±0.31**^**a**^	**3.02±0.22**^**b**^	**23.22±0.85**^**a**^	**19.74±0.92**^**b**^
***P*-value**			<0.0001*		<0.0001*		<0.0001*	

FA & N-FA denotes Favourable and Non-favourable alleles respectively.

^#^highly favourable alleles that exhibit significantly different traits compared with the unfavorable alleles (P < 0.05)

*P* values are for comparison between mean GL and GLWR values of cumulated FAs and N-FAs

*ANOVA test for significant at the level of *P* value <0.0001.

^a^ and ^b^ were ranked by Tukey (HSD) test

### Genetic diversity

A total of ten markers from 7 grain size related genes were used to assess the genetic diversity of 89 rice germplasm. The major allele frequency varied from 0.50 to 0.92 with an average of 0.7. The polymorphism information content of markers had an average value of 0.31 and varied from 0.13 to 0.58. The maximum PIC value was observed for the marker GS3-*Pst*I of *GS3* gene while minimum for the marker RM574 of *GS5* gene. Similarly, the gene diversity of ten markers ranged from 0.14 to 0.66 with a mean value of 0.39 (Table 6). The present study showed that the GS3-*Pst*1 of *GS3* gene is highly informative for grain size and can be used to assess the genetic diversity of diverse germplasm whereas rest of the markers are moderate to slightly informative.

**Table 6:** Estimation of major allele frequency, allele per locus, gene diversity, expected heterozygosity, PIC in 89 rice germplasm.

Marker	Major Allele Frequency	Allele per locus	Gene Diversity	Expected heterozygosity	PIC
S9	0.505	2	0.499	0.499	0.375
FMGS7	0.853	2	0.249	0.393	0.316
GS3-*Pst*I	0.370	3	0.660	0.660	0.586
RSG-1	0.651	2	0.453	0.454	0.350
GW8-InDel	0.752	2	0.372	0.372	0.302
RID76	0.730	2	0.393	0.393	0.316
RID711	0.696	2	0.422	0.422	0.333
RM574	0.921	2	0.144	0.144	0.134
W004	0.730	2	0.393	0.393	0.316
SR17	0.853	2	0.249	0.249	0.218
**Mean**	**0.706**	**2.1**	**0.384**	**0.398**	**0.324**

**Fig 4:**
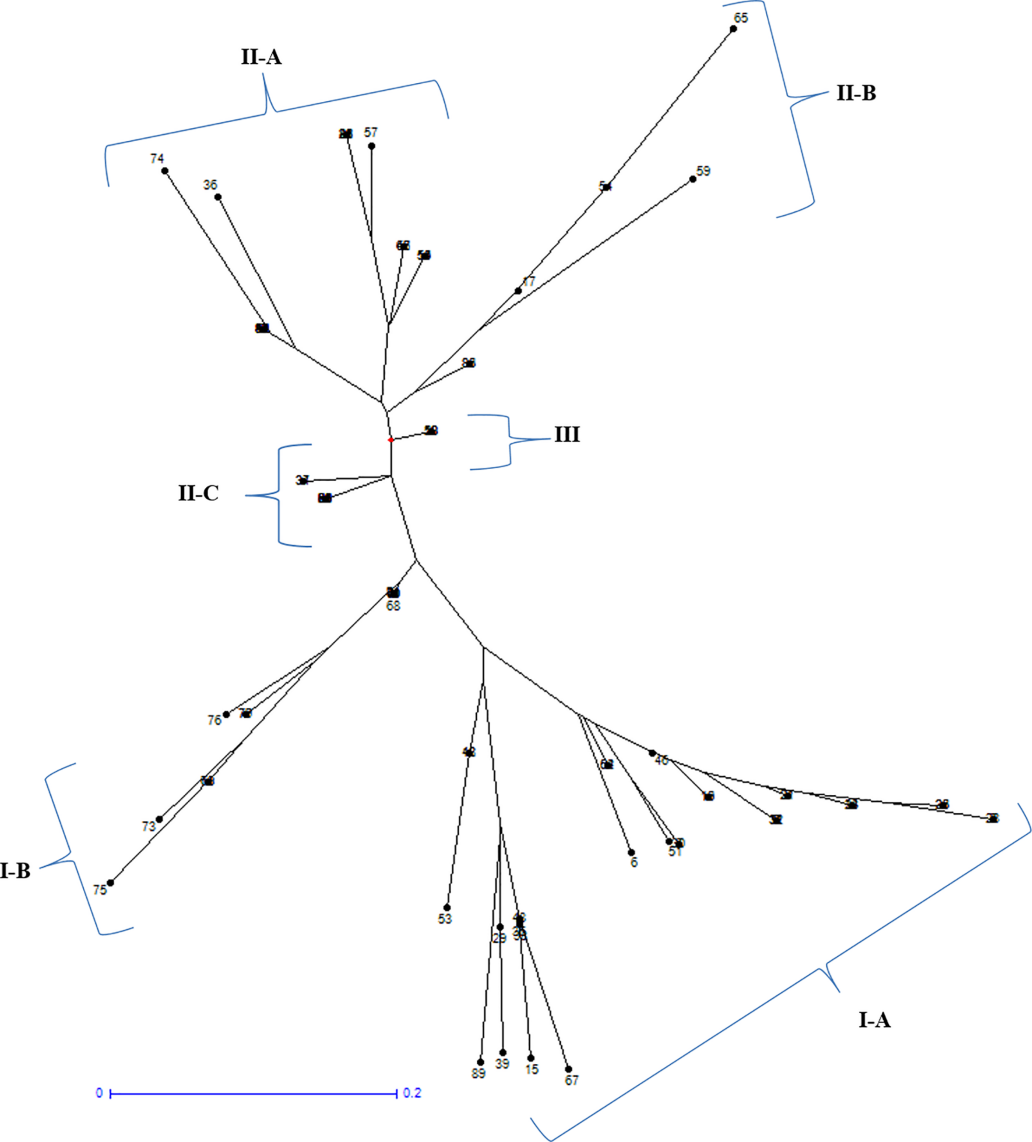
Cluster analysis of 89 germplasm based on 7 grain size regulating genes.

Based on cluster analysis, 89 germplasm were categorized into three major clusters (I, II and III) **(**Fig 4). Major cluster I included 29 germplasm, which was further divided into two sub-clusters IA and IB; the sub-cluster IA and IB consists of 18 and 11 germplasm respectively. The mean grain length of major cluster I was observed to be 7.83 mm and mean grain width was 2.44 mm. Major cluster II contained 54 germplasm of which most of them belonged to medium rice grain with the mean grain length and width of 8.2 and 2.4 mm, respectively. Major cluster III included only 6 germplasm with a mean grain length of 9.38 mm.

### Genetic association of seven genes for grain size

Genetic association analysis of the 7 selected genes with the grain length, width, GLWR and TGW traits were carried out using the generalized linear model (GLM) to find out significant association among GL, GW, GLWR and TGW. Out of the ten markers used in the present study, only one marker RID711 of *GL7* gene was significantly associated with all the four traits viz. GL, GW, GLWR and TGW (Table 7). The GS3-*Pst*I marker for *GS3* gene showed the highest phenotypic variance (44.2%) followed by FMGS7 (25.8%), RID76 (14%), GW8-InDel (11%) and RID76 (RID711 (10.4%). Out of the ten markers, only seven markers (S9, FMGS7, GS3-*Pst*I, GW8-indel, RID76, RID711 and RM574) corresponding to six genes (*DEP1*, *GS7*, *GS3*, *GW8*, *GL7* and *GS5*) showed significant association for GL with a range of phenotypic variation from 4.9 to 44.2%. The GS3-*Pst*I marker for *GS3* gene showed the highest phenotypic variance (44.2%) and RM574 of *GS5* genes showed the lowest (4.9%). The other three markers for grain length didn’t reveal significant association at the level of *P* value < 0.1. Similar associations of the same markers were also observed during the analysis of variance.

**Table 7:** Genetic association of 7 genes for rice grain size traits.

		Grain length (GL)		Grain width (GW)		GLWR		TGW	
S.N.	Marker	*P*-value	R^2^_marker	*P*-value	R^2^_marker	*P*-value	R^2^_marker	*P*-value	R^2^_marker
1	S9	0.007*	0.078	0.575	0.003	0.013*	0.068	0.00591**	0.08386
2	FMGS7	3.E-07**	0.258	0.238	0.016	4.E-06**	0.215	0.00389**	0.09183
3	GS3-*Pst*I	1.E-12**	0.442	0.085	0.034	1.E-11**	0.412	9.46E-05**	0.16153
4	RSG1	0.458	0.006	0.0203*	0.061	0.289	0.012	0.68097	0.00195
5	GW8-InDel	0.001**	0.110	0.019*	0.062	0.003*	0.092	0.23757	0.016
6	RID76	2.E-04**	0.140	0.560	0.004	7.E-04**	0.122	0.01441*	0.06688
7	RID711	0.002*	0.104	0.018*	0.063	0.006*	0.081	0.01069*	0.07256
8	RM574	0.037*	0.049	0.042*	0.047	0.090	0.032	0.349	0.01009
9	W004	0.080	0.034	0.700	0.001	0.088	0.033	0.77768	9.21E-04
10	SR17	0.959	3.E-05	0.121	0.028	0.793	7.E-04	0.58758	0.00339

*& * * significant at *P* value <0.05, <0.001 respectively

Similarly, four markers were found to be associated with the grain width i.e. RSG1, GW8-InDel, RID711, and RM574. The phenotypic variance for these four markers for grain width varied from 4.7% to 6.3%, respectively. The marker RID711 represented highest phenotypic variance (6.3%) followed by GW8-InDel (6.2%), RSG1 (6.1%), and RM574 (4.7%) whereas, remaining six markers for grain width didn’t reveal significant association at *P* < 0.05.

In the case of GLWR, only six markers were found to be significantly associated with the GLWR at *P* < 0.05. These markers (S9, FMGS7, GW8-InDel, RID711 RM574 and W004) represented a phenotypic variance from 6.8% to 41.2%. The GS3-*Pst*I marker for *GS3* gene showed the highest phenotypic variance (41.2%) and S9 of *DEP1* genes showed the lowest (6.8%). Interestingly, except RM574, all the markers associated with GL were also found to be significantly associated with GLWR. This is another evidence for the hypothesis where the GL contributes major effects to GLWR as compared to GW.

Except for RM574 and GW8-InDel, all the remaining five markers showing significant association with GL and GLWR were found to be significantly associated with TGW in 89 germplasm. The phenotypic variance for these five markers for grain width varied from 6% to 16.1%.

### Population structure analysis

**Fig 5:**
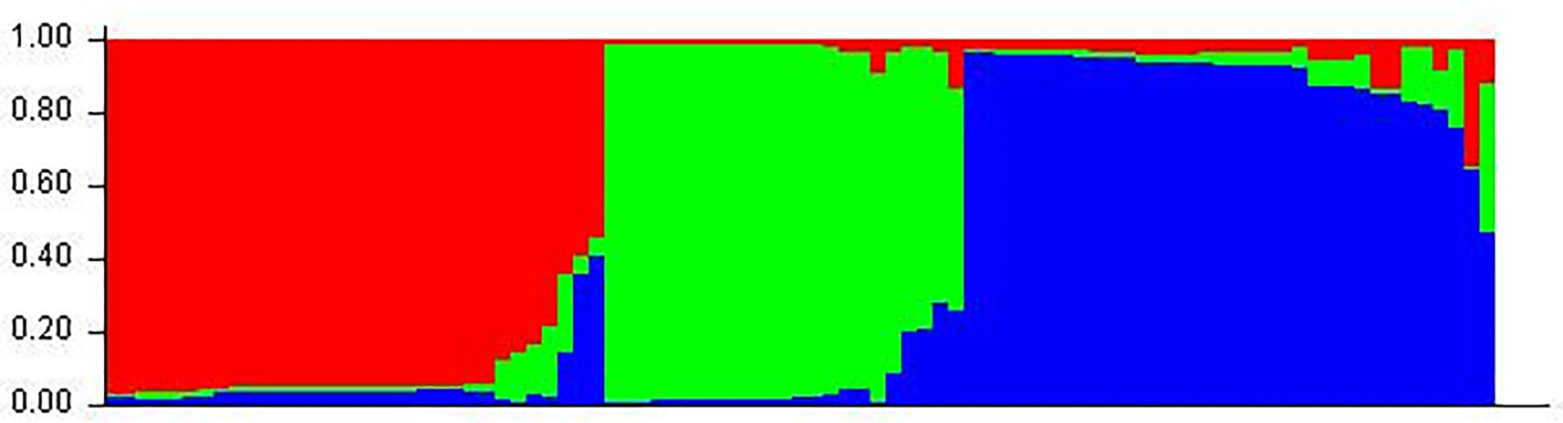
Population structure of 89 germplasm based on 10 markers (K = 3) and graph of estimated membership fraction for K = 3. The maximum of adhoc measure ΔK determined by structure harvester was found to be K = 3, which indicated that the entire population can be grouped into three subgroups. Different color within group indicates the proportion of shared ancestry with other group which has the same color with the admixture.

Population structure of 89 rice germplasm was analysed using the data obtained from eleven markers using Structure software. The peak plateau of *adhoc* measure ΔK was found to be K = 3 (Fig 5) which indicated that the entire 89 germplasm were distributed into three subgroups (SG1 SG2 and SG3). Based on ancestry threshold of >55%, all the 89 germplasm except two, were classified into three subgroups (S2 Table). The SG1 contain 31 germplasm, most of which belonged to medium grain (17), while 10 and 4 belong to long and short grain respectively. The SG2 included all the grain type, i.e. small (8), medium (14) and long grain (3) types. Further, the SG3 subgroup is dominated by the long grain type germplasm (25) of 31 germplasm. Notably, each sub-group is dominated by a particular grain type like SG1 and SG2 (medium grain type), and SG3 (long grain). The result revealed that the subgroup SG3 was considered dominated by long grain type whereas the subgroup SG1 and SG2 mostly consisted of medium grain type. Consequently, as a whole, structure analysis suggested the differentiation of grain type in three separate populations (SG1, SG2 and SG3).

### Estimation of population genetics through AMOVA analysis

**Fig 6:**
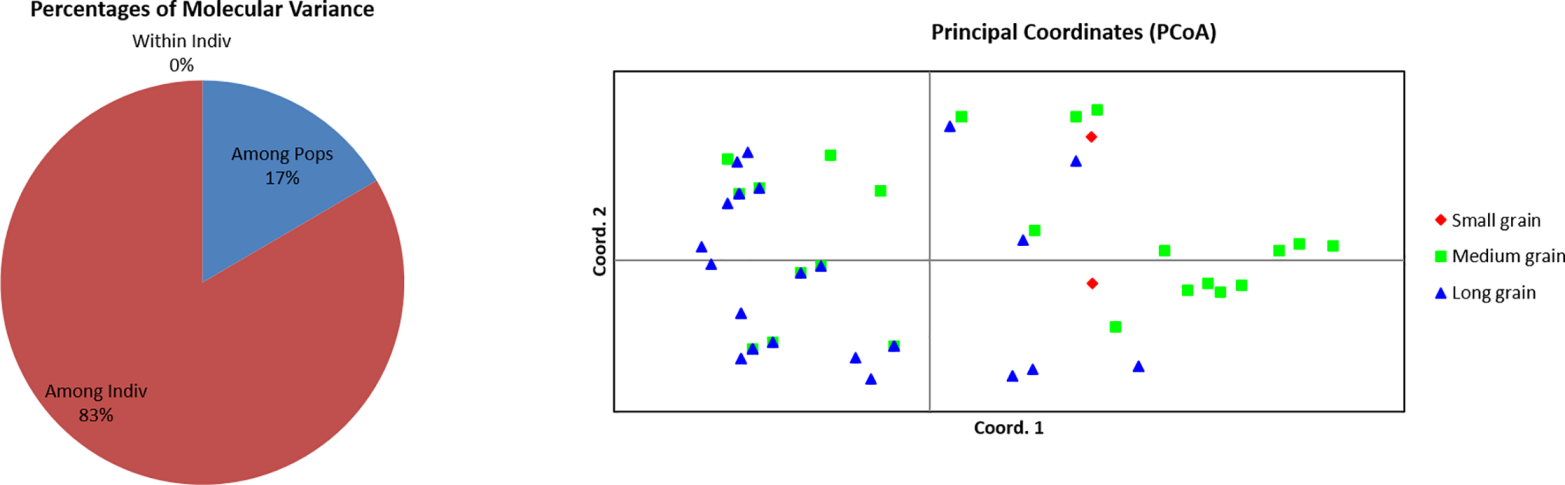
Analysis of molecular variance (AMOVA) and Principal Coordinate Analysis (PCoA) of 89 rice germplasm based on 10 linked/functional markers. Two dimensional PCoA display of 89 germplasm based on 10 markers. Coord-1 and Coord-2 represent first and second coordinates, respectively. The two PCA axes accounted for 32.89% and 20.98% % of the genetic variation among populations.

In order to find out the genetic relationship among the three grain type populations, the 89 rice germplasm were divided into three groups based on grain size; Small grain (length <6 mm, 14 germplasm); medium grain (length 6–8 mm; 35 germplasm) and long grain (length > 9 mm; 40 germplasm). It was found that maximum variance (83%) was observed within the population while minimum exist between populations (17%) (Fig 6, Table 8). The pair-wise fixation indices (F_ST_) among the populations were given in the Table 9. The highest pair wise F_ST_ was observed between small grain and long grain rice while the least was observed between small and medium grain types. The estimated value of fixation indices indicated that there is weak population structure which are not genetically isolated from each other. To establish the genetic relationships of the 89 germplasm based on the ten markers related to seven genes, the PCoA was further constructed. A scatter plot generated from the PCoA analysis showed that the first two components accounted to 32.89% and 20.98% of the genetic variation which resulted in a total genetic variation of 53.87% (Fig 6). These scatter plots showed a clear separation of two grain types, long and medium grain populations. This result is found to be concurrent with the result of the clustered and structure analysis.

**Table 8:** Results of analysis of molecular variance (AMOVA).

Source	df	SS	MS	Est. Var.	%
**Among Pops**	2	42.311	21.155	0.325	17%
**Among Individual**	86	281.150	3.269	1.635	83%
**Within Indiv**	89	0.000	0.000	0.000	0%
**Total**	177	323.461		1.959	100%

Df: degree of freedom; SS; sum of squares, Est. Var.: Estimated variance

**Table 9:** Pair-wise F_ST_ estimates among three populations of germplasm.

	Small grain	Medium grain	Long grain
**Small grain**	0.000		
**Medium grain**	0.014	0.000	
**Long grain**	0.272	0.168	0.000

F_ST_ values below diagonal.

## Conclusion

The effects of different allelic combinations from different genes to determine the final grain shape and size undertaken in this study would further facilitate understanding the complex mechanism of rice grain size. Our finding provided an overview of the allelic combinations of different genes for grain size in rice. The beneficial alleles identified could be useful to manipulate and pyramid the preferred grain size trait for consumer’s interest thereby increase the grain yield. Besides, the marker loci that strongly associated with grain size would be highly informative and efficient in the selection of parental lines for development of new rice breeding populations.

## Supporting information

S1 Table Phenotypic data of rice germplasm used in the present study. (DOCX)

S2 Table Population structure groups of rice germplasm based on inferred ancestry values. (DOCX)

## References

[1] Ngangkham U, Parida SK, De S, Kumar KA, Singh AK, Singh NK, et al Genic markers for wild abortive (WA) cytoplasm based male sterility and its fertility restoration in rice. Mol Breeding. 2010; 26(2): 275–92.

[2] Huang X, Zhao Y, Li C, Wang A, Zhao Q, Li W, et al Genome-wide association study of flowering time and grain yield traits in a worldwide collection of rice germplasm. Nat Genet. 2012; 44(1): 32–9. 10.1038/ng.1018 22138690

[3] Lan L, Chen W, Lai Y, Suo J, Kong Z, Li C, et al Monitoring of gene expression profiles and isolation of candidate genes involved in pollination and fertilization in rice (*Oryza sativa* L.) with a 10K cDNA microarray. Plant Mol Biol. 2004; 54(4): 471–87. doi: 10.1023/B:PLAN.0000038254.58491.c7 15316284 10.1023/B:PLAN.0000038254.58491.c7

[4] Tang J, Li D, Cao M, Tao Y, Tong W, Zhang X, et al Gene expression profiling in rice young panicle and vegetative organs and identification of panicle-specific genes through known gene functions. Mol Genet Genomics. 2005; 274(5): 467–76. doi: 10.1007/s00438-005-0043-2 16211393 10.1007/s00438-005-0043-2

[5] Thomas WT. Prospects for molecular breeding of barley. Annals of Applied Biology. 2003; 142(1): 1–2.

[6] Doi K, Yasui H, Yoshimura A. Genetic variation in rice. Curr Opin Plant Biol. 2008; 11(2): 144–8. doi: 10.1016/j.pbi.2008.01.008 18316240 10.1016/j.pbi.2008.01.008

[7] Fan C, Xing Y, Mao H, Lu T, Han B, Xu C, et al GS3, a major QTL for grain length and weight and minor QTL for grain width and thickness in rice, encodes a putative transmembrane protein. Theor Appl Genet. 2006; 112(6): 1164–71. doi: 10.1007/s00122-006-0218-1 16453132 10.1007/s00122-006-0218-1

[8] Mao H, Sun S, Yao J, Wang C, Yu S, Xu C, et al Linking differential domain functions of the GS3 protein to natural variation of grain size in rice. Proc Natl Acad Sci U S A. 2010; 107(45): 19579–84. doi: 10.1073/pnas.1014419107 20974950 10.1073/pnas.1014419107 PMC2984220

[9] Xian-Jun S, Huang W, Shi M, Zhu MZ, Hong-Xuan L. A QTL for rice grain width and weight encodes a previously unknown RING-type E3 ubiquitin ligase. Nat Genet. 2007; 39(5): 623 doi: 10.1038/ng2014 17417637 10.1038/ng2014

[10] Shomura A, Izawa T, Ebana K, Ebitani T, Kanegae H, Konishi S, et al Deletion in a gene associated with grain size increased yields during rice domestication. Nat Genet. 2008; 40(8): 1023–8. doi: 10.1038/ng.169 18604208 10.1038/ng.169

[11] Weng J, Gu S, Wan X, Gao H, Guo T, Su N, et al Isolation and initial characterization of GW5, a major QTL associated with rice grain width and weight. Cell Res. 2008; 18(12): 1199 doi: 10.1038/cr.2008.307 19015668 10.1038/cr.2008.307

[12] Li Y, Fan C, Xing Y, Jiang Y, Luo L, Sun L, et al Natural variation in GS5 plays an important role in regulating grain size and yield in rice. Nat Genet. 2011; 43(12): 1266–9. doi: 10.1038/ng.977 22019783 10.1038/ng.977

[13] Qi P, Lin YS, Song XJ, Shen JB, Huang W, Shan JX, et al The novel quantitative trait locus GL3. 1 controls rice grain size and yield by regulating Cyclin-T1; 3. Cell Res. 2012; 22(12): 1666 doi: 10.1038/cr.2012.151 23147796 10.1038/cr.2012.151 PMC3515756

[14] Zhang X, Wang J, Huang J, Lan H, Wang C, Yin C, et al Rare allele of OsPPKL1 associated with grain length causes extra-large grain and a significant yield increase in rice. Proc Natl Acad Sci U S A. 2012; 109(52): 21534–9. doi: 10.1073/pnas.1219776110 23236132 10.1073/pnas.1219776110 PMC3535600

[15] Wang S, Wu K, Yuan Q, Liu X, Liu Z, Lin X, et al Control of grain size, shape and quality by OsSPL16 in rice. Nat Genet. 2012; 44(8): 950 doi: 10.1038/ng.2327 22729225 10.1038/ng.2327

[16] Ishimaru K, Hirotsu N, Madoka Y, Murakami N, Hara N, Onodera H, et al Loss of function of the IAA-glucose hydrolase gene TGW6 enhances rice grain weight and increases yield. Nat Genet. 2013; 45(6): 707–11. doi: 10.1038/ng.2612 23583977 10.1038/ng.2612

[17] Wang Y, Xiong G, Hu J, Jiang L, Yu H, Xu J, et al Copy number variation at the GL7 locus contributes to grain size diversity in rice. Nat Genet. 2015; 47(8): 944–8. doi: 10.1038/ng.3346 26147619 10.1038/ng.3346

[18] Wang S, Li S, Liu Q, Wu K, Zhang J, Wang S, et al The OsSPL16-GW7 regulatory module determines grain shape and simultaneously improves rice yield and grain quality. Nat Genet. 2015; 47(8): 949–54. doi: 10.1038/ng.3352 26147620 10.1038/ng.3352

[19] Ashikari M, Sakakibara H, Lin S, Yamamoto T, Takashi T, Nishimura A, et al Cytokinin oxidase regulates rice grain production. Science. 2005; 309(5735): 741–5. doi: 10.1126/science.1113373 15976269 10.1126/science.1113373

[20] Huang X, Qian Q, Liu Z, Sun H, He S, Luo D, et al Natural variation at the DEP1 locus enhances grain yield in rice. Nat Genet. 2009; 41(4): 494–7. doi: 10.1038/ng.352 19305410 10.1038/ng.352

[21] Jiao Y, Wang Y, Xue D, Wang J, Yan M, Liu G, et al Regulation of OsSPL14 by OsmiR156 defines ideal plant architecture in rice. Nat Genet. 2010; 42(6): 541–4. doi: 10.1038/ng.591 20495565 10.1038/ng.591

[22] Miura K, Ikeda M, Matsubara A, Song XJ, Ito M, Asano K, et al OsSPL14 promotes panicle branching and higher grain productivity in rice. Nat Genet. 2010; 42(6): 545–9. doi: 10.1038/ng.592 20495564 10.1038/ng.592

[23] Wang E, Wang J, Zhu X, Hao W, Wang L, Li Q, et al Control of rice grain-filling and yield by a gene with a potential signature of domestication. Nat Genet. 2008; 40(11): 1370–4. doi: 10.1038/ng.220 18820698 10.1038/ng.220

[24] Zuo J, Li J. Molecular genetic dissection of quantitative trait loci regulating rice grain size. Annu Rev Genet. 2014; 48: 99–118. doi: 10.1146/annurev-genet-120213-092138 25149369 10.1146/annurev-genet-120213-092138

[25] Perrier X, Jacquemoud-Collet JP. DARwin software. 2006. Available from: http://darwin.cirad.fr/darwin.

[26] Jan SJK. PIC calculator. 2002. Available from: http://www.liv.ac.uk/~kempsj/pic.html.

[27] Lui K, Muse SV. PowerMarker: integrated analysis environment for genetic marker data. Bioinformatics. 2005; 21(9): 2128–9. doi: 10.1093/bioinformatics/bti282 15705655 10.1093/bioinformatics/bti282

[28] Bradbury PJ, Zhang Z, Kroon DE, Casstevens TM, Ramdoss Y, Buckler ES. TASSEL: software for association mapping of complex traits in diverse samples. Bioinformatics. 2007; 23(19): 2633–5. doi: 10.1093/bioinformatics/btm308 17586829 10.1093/bioinformatics/btm308

[29] Pritchard JK, Stephens M, Donnelly P. Inference of population structure using multilocus genotype data. Genetics. 2000; 155(2): 945–59. 10835412 10.1093/genetics/155.2.945 PMC1461096

[30] Evanno G, Regnaut S, Goudet J. Detecting the number of clusters of individuals using the software STRUCTURE: a simulation study. Mol Ecol. 2005; 14(8):2611–20. doi: 10.1111/j.1365-294X.2005.02553.x 15969739 10.1111/j.1365-294X.2005.02553.x

[31] Earl DA. STRUCTURE HARVESTER: a website and program for visualizing STRUCTURE output and implementing the Evanno method. Conservation Genetics Resources. 2012; 4(2): 359–61.

[32] Peakall RO, Smouse PE. GENALEX 6: genetic analysis in Excel. Population genetic software for teaching and research. Molecular Ecology Notes. 2006; 6(1): 288–295. 10.1093/bioinformatics/bts460 PMC3463245 22820204

[33] Zhu T, Budworth P, Chen W, Provart N, Chang HS, Guimil S, et al Transcriptional control of nutrient partitioning during rice grain filling. Plant Biotechnol J. 2003; 1(1): 59–70. doi: 10.1046/j.1467-7652.2003.00006.x 17147681 10.1046/j.1467-7652.2003.00006.x

[34] Koutroubas SD, Mazzini F, Pons B, Ntanos DA. Grain quality variation and relationships with morpho-physiological traits in rice (*Oryza sativa* L.) genetic resources in Europe. Field Crops Res. 2004; 86(2): 115–30.

[35] Dalla Corte A, Moda-Cirino V, Arias CA, Toledo JF, Destro D. Genetic analysis of seed morphological traits and its correlations with Grain yield in common bean. Braz Arch Biol Technol. 2010; 53(1): 27–34.

[36] Kitagawa K, Kurinami S, Oki K, Abe Y, Ando T, Kono I, et al A novel kinesin 13 protein regulating rice seed length. Plant and Cell Physiol. 2010; 51(8): 1315–29. 20587735 10.1093/pcp/pcq092

[37] Lu L, Shao D, Qiu X, Sun L, Yan W, Zhou X, et al Natural variation and artificial selection in four genes determine grain shape in rice. New Phytol. 2013; 200(4): 1269–80. doi: 10.1111/nph.12430 23952103 10.1111/nph.12430

[38] Fan C, Xing Y, Mao H, Lu T, Han B, Xu C, et al GS3, a major QTL for grain length and weight and minor QTL for grain width and thickness in rice, encodes a putative transmembrane protein. Theor Appl Genet. 2006; 112(6): 1164–71. doi: 10.1007/s00122-006-0218-1 16453132 10.1007/s00122-006-0218-1

[39] Yan S, Zou G, Li S, Wang H, Liu H, Zhai G, et al Seed size is determined by the combinations of the genes controlling different seed characteristics in rice. Theor Appl Genet. 2011; 123(7): 1173 doi: 10.1007/s00122-011-1657-x 21805338 10.1007/s00122-011-1657-x

[40] Fan C, Yu S, Wang C, Xing Y. A causal C–A mutation in the second exon of GS3 highly associated with rice grain length and validated as a functional marker. Theor Appl Genet. 2009; 118(3): 465–72. doi: 10.1007/s00122-008-0913-1 19020856 10.1007/s00122-008-0913-1

[41] Takano-Kai N, Jiang H, Kubo T, Sweeney M, Matsumoto T, Kanamori H, et al Evolutionary history of GS3, a gene conferring grain length in rice. Genetics. 2009; 182(4): 1323–34. doi: 10.1534/genetics.109.103002 19506305 10.1534/genetics.109.103002 PMC2728869

[42] Lee CM, Park J, Kim B, Seo J, Lee G, Jang S, et al Influence of multi-gene allele combinations on grain size of rice and development of a regression equation model to predict grain parameters. Rice. 2015; 8(1): 33 doi: 10.1186/s12284-015-0066-1 26519289 10.1186/s12284-015-0066-1 PMC4627975

[43] Wang C, Chen S, Yu S. Functional markers developed from multiple loci in GS3 for fine marker-assisted selection of grain length in rice. Theor Appl Genet. 2011; 122(5): 905–13. doi: 10.1007/s00122-010-1497-0 21107518 10.1007/s00122-010-1497-0

[44] Takano-Kai N, Jiang H, Powell A, McCouch S, Takamure I, Furuya N, et al Multiple and independent origins of short seeded alleles of GS3 in rice. Breed Sci. 2013; 63(1): 77–85. doi: 10.1270/jsbbs.63.77 23641184 10.1270/jsbbs.63.77 PMC3621448

[45] Sironen A, Thomsen B, Andersson M, Ahola V, Vilkki J. An intronic insertion in KPL2 results in aberrant splicing and causes the immotile short-tail sperm defect in the pig. Proc Natl Acad Sci U S A. 2006; 103(13): 5006–11. doi: 10.1073/pnas.0506318103 16549801 10.1073/pnas.0506318103 PMC1458785

[46] Kim SR, Ramos J, Ashikari M, Virk PS, Torres EA, Nissila E, et al Development and validation of allele-specific SNP/indel markers for eight yield-enhancing genes using whole-genome sequencing strategy to increase yield potential of rice, *Oryza sativa* L. Rice. 2016; 9(1): 12 doi: 10.1186/s12284-016-0084-7 26987543 10.1186/s12284-016-0084-7 PMC4797370

[47] Dixit N, Dokku P, Mithra SA, Parida SK, Singh AK, Singh NK, et al Haplotype structure in grain weight gene GW2 and its association with grain characteristics in rice. Euphytica. 2013; 192(1): 55–61.

[48] Shao G, Wei X, Chen M, Tang S, Luo J, Jiao G, et al Allelic variation for a candidate gene for GS7, responsible for grain shape in rice. Theor Appl Genet. 2012; 125(6): 1303–12. doi: 10.1007/s00122-012-1914-7 22772587 10.1007/s00122-012-1914-7

[49] Li W, Zhang B, Li R, Chang X, Jing R. Favourable alleles for stem water-soluble carbohydrates identified by association analysis contribute to grain weight under drought stress conditions in wheat. PLoS One. 2015; 10(3): e0119438 doi: 10.1371/journal.pone.0119438 25768726 10.1371/journal.pone.0119438 PMC4358973

