# Supplementary material for: Effect of multiple allelic combinations of genes on regulating grain size in rice
Source: PLoS One. 2018 Jan 5;13(1):e0190684. doi: 10.1371/journal.pone.0190684 (PMC5755915; doi:10.1371/journal.pone.0190684)
Supplement: S1 Table — (DOCX) [file pone.0190684.s001.docx]

**S1 Table. Phenotypic data of rice germplasm used in the present study**

| **Sl No.** | **Germplasm** | **Grain length (mm)** | **Grain width (mm)** | **Grain length-width ratio** | **Grain weight (g)** |
| --- | --- | --- | --- | --- | --- |
| 1 | IC46047 | 5.07 | 1.85 | 2.74 | 11.3 |
| 2 | IC343547 | 5.23 | 1.9 | 2.75 | 10.5 |
| 3 | IC386761 | 5.37 | 1.94 | 2.77 | 11.1 |
| 4 | IC399092 | 5.41 | 2.57 | 2.11 | 10.6 |
| 5 | IC122114 | 5.42 | 1.85 | 2.93 | 9.3 |
| 6 | IC463145 | 5.43 | 1.99 | 2.73 | 21.5 |
| 7 | IC200542 | 5.62 | 1.75 | 3.21 | 8.7 |
| 8 | IC40788 | 5.62 | 3.17 | 1.77 | 16.1 |
| 9 | IC454353 | 5.63 | 1.91 | 2.95 | 13.3 |
| 10 | IC390656 | 5.72 | 2.41 | 2.37 | 15.8 |
| 11 | MGR-1 | 5.73 | 2.93 | 1.96 | 16.2 |
| 12 | IC21774 | 5.79 | 2.25 | 2.57 | 20.8 |
| 13 | IC39743 | 5.89 | 2.81 | 2.10 | 17.3 |
| 14 | IC5999 | 5.94 | 2.56 | 2.32 | 16.9 |
| 15 | SR39 | 6.09 | 2.39 | 2.55 | 21.8 |
| 16 | VTL505 | 6.18 | 3 | 2.06 | 22.9 |
| 17 | IC390554 | 6.28 | 3.01 | 2.09 | 18.5 |
| 18 | IC35107 | 6.29 | 2.74 | 2.30 | 14.7 |
| 19 | IC451235 | 6.37 | 2.35 | 2.71 | 23.1 |
| 20 | Tsamumfiirai | 6.41 | 2.32 | 2.76 | 12.2 |
| 21 | IC181 | 6.41 | 2.55 | 2.51 | 26 |
| 22 | IC39849 | 6.42 | 2.33 | 2.76 | 23.3 |
| 23 | IC38098 | 6.42 | 2.25 | 2.85 | 22.4 |
| 24 | IC86581 | 6.43 | 2.28 | 2.82 | 25.7 |
| 25 | IC25867 | 6.51 | 2.9 | 2.24 | 16.1 |
| 26 | Eypo | 6.58 | 3.12 | 2.11 | 18.5 |
| 27 | IC74782 | 6.6 | 2.23 | 2.96 | 26.5 |
| 28 | IC74717 | 6.62 | 2.08 | 3.18 | 27.8 |
| 29 | IC67706 | 6.63 | 2.3 | 2.88 | 13.4 |
| 30 | IC52785 | 6.66 | 2.79 | 2.39 | 12.6 |
| 31 | IC67730 | 6.66 | 3.35 | 1.99 | 19.9 |
| 32 | Tsukji | 6.71 | 2.42 | 2.77 | 16.5 |
| 33 | IC115954 | 6.72 | 2.85 | 2.36 | 17.6 |
| 34 | IC12168 | 6.72 | 2.63 | 2.56 | 17.2 |
| 35 | IC74773 | 6.74 | 2.63 | 2.56 | 15.9 |
| 36 | IC36753 | 6.75 | 2.31 | 2.92 | 20.4 |
| 37 | IC134972 | 6.82 | 3.15 | 2.17 | 27.8 |
| 38 | IC8960 | 6.82 | 2.73 | 2.50 | 24.7 |
| 39 | IC27513 | 6.85 | 2.65 | 2.58 | 26.1 |
| 40 | IC330458 | 6.85 | 2.59 | 2.64 | 21.6 |
| 41 | IC35552 | 6.89 | 2.66 | 2.59 | 25.8 |
| 42 | IC45701 | 6.89 | 1.71 | 4.03 | 15.6 |
| 43 | IC19981 | 6.9 | 2.27 | 3.04 | 23.3 |
| 44 | IC66819 | 6.9 | 2.2 | 3.14 | 26.9 |
| 45 | IC209047 | 6.91 | 2.62 | 2.64 | 21.5 |
| 46 | IC12166 | 6.93 | 2.59 | 2.68 | 15.5 |
| 47 | IC145758 | 6.93 | 2.23 | 3.11 | 24.6 |
| 48 | IC17042 | 6.93 | 2.14 | 3.24 | 20 |
| 49 | IC35214 | 6.95 | 2.66 | 2.61 | 27.9 |
| 50 | IC25850 | 9.87 | 2.33 | 4.24 | 28.1 |
| 51 | BAM7244 | 9.96 | 2.19 | 4.55 | 25.6 |
| 52 | IC6294 | 10.07 | 2.07 | 4.86 | 19.9 |
| 53 | Jalmagan | 10.09 | 2.36 | 4.28 | 24.1 |
| 54 | IC208092 | 10.12 | 2.49 | 4.06 | 22.1 |
| 55 | IC36704 | 10.12 | 1.91 | 5.30 | 29.3 |
| 56 | BAM8030 | 10.13 | 2.5 | 4.05 | 29.6 |
| 57 | Desitian Raj | 10.13 | 2.05 | 4.94 | 28 |
| 58 | IC73121 | 10.13 | 2.26 | 4.48 | 27.3 |
| 59 | BAM7607 | 10.23 | 2.57 | 3.98 | 28.5 |
| 60 | BAM7482 | 10.24 | 2.54 | 4.03 | 25.1 |
| 61 | IC116983 | 10.28 | 2.4 | 4.28 | 26.8 |
| 62 | IC342630 | 10.3 | 2.15 | 4.79 | 24.8 |
| 63 | BAM8032 | 10.32 | 2.22 | 4.65 | 23.9 |
| 64 | IC8177 | 10.38 | 2.42 | 4.29 | 27.5 |
| 65 | CSR30 | 10.43 | 2.08 | 5.01 | 23.3 |
| 66 | IC418922 | 10.51 | 2.11 | 4.98 | 17.4 |
| 67 | BAM7354 | 10.57 | 2.12 | 4.99 | 18.9 |
| 68 | IC8506 | 10.58 | 2.03 | 5.21 | 16.4 |
| 69 | CSR8 | 10.71 | 1.95 | 5.49 | 19.8 |
| 70 | Gujarat5 | 10.81 | 1.98 | 5.46 | 22.5 |
| 71 | IC116989 | 10.86 | 2.83 | 3.84 | 28.8 |
| 72 | IC377621 | 11.23 | 2.47 | 4.55 | 23.4 |
| 73 | Azucena | 11.24 | 2.68 | 4.19 | 28.3 |
| 74 | Camponi sml | 11.48 | 2.4 | 4.78 | 29.7 |
| 75 | IC346909 | 11.91 | 2.17 | 5.49 | 28.4 |
| 76 | BAM8070 | 12.3 | 2.32 | 5.30 | 19.7 |
| 77 | BAM8069 | 12.48 | 2.26 | 5.52 | 29.8 |
| 78 | BAM8068 | 12.78 | 2.3 | 5.56 | 26.4 |
| 79 | Do Dou | 9.75 | 2.08 | 4.69 | 22.8 |
| 80 | IC123723 | 9.76 | 3.02 | 3.23 | 21 |
| 81 | IC4434 | 9.76 | 2.64 | 3.70 | 27.1 |
| 82 | IC137492 | 9.78 | 2.78 | 3.52 | 23.5 |
| 83 | BAM8076 | 9.79 | 2.2 | 4.45 | 17.4 |
| 84 | PR116 | 9.79 | 1.95 | 5.02 | 19.9 |
| 85 | BAM8031 | 9.9 | 2.06 | 4.81 | 24.5 |
| 86 | IC38062 | 9.91 | 2.32 | 4.27 | 28.2 |
| 87 | IC61953 | 9.92 | 2.72 | 3.65 | 24.5 |
| 88 | IC253851 | 9.93 | 2.5 | 3.97 | 26.5 |
| 89 | BAM7708 | 9.95 | 2.15 | 4.63 | 24.8 |
