# Supplementary material for: Effect of multiple allelic combinations of genes on regulating grain size in rice
Source: PLoS One. 2018 Jan 5;13(1):e0190684. doi: 10.1371/journal.pone.0190684 (PMC5755915; doi:10.1371/journal.pone.0190684)
Supplement: S2 Table — (DOCX) [file pone.0190684.s002.docx]

**S2 Table. Population structure groups of rice germplasm based on Inferred ancestry values**

| S.No. | Germplasm | Q1 | Q2 | Q3 |
| --- | --- | --- | --- | --- |
| 1 | IC46047 | 0.031 | 0.923 | 0.046 |
| 2 | IC343547 | 0.054 | 0.069 | 0.877 |
| 3 | IC386761 | 0.013 | 0.972 | 0.015 |
| 4 | IC399092 | 0.034 | 0.874 | 0.092 |
| 5 | IC122114 | 0.012 | 0.972 | 0.016 |
| 6 | IC463145 | 0.947 | 0.013 | 0.04 |
| 7 | IC200542 | 0.087 | 0.901 | 0.012 |
| 8 | IC40788 | 0.943 | 0.013 | 0.044 |
| 9 | IC454353 | 0.013 | 0.972 | 0.015 |
| 10 | IC390656 | 0.642 | 0.214 | 0.144 |
| 11 | MGR-1 | 0.011 | 0.972 | 0.016 |
| 12 | IC21774 | 0.08 | 0.111 | 0.809 |
| 13 | IC39743 | 0.784 | 0.194 | 0.022 |
| 14 | IC5999 | 0.012 | 0.969 | 0.019 |
| 15 | SR39 | 0.965 | 0.01 | 0.025 |
| 16 | VTL505 | 0.055 | 0.069 | 0.876 |
| 17 | IC390554 | 0.013 | 0.968 | 0.019 |
| 18 | IC35107 | 0.053 | 0.069 | 0.877 |
| 19 | IC451235 | 0.014 | 0.963 | 0.023 |
| 20 | TSAMUMFIIRAI | 0.948 | 0.015 | 0.037 |
| 21 | IC181 | 0.01 | 0.98 | 0.01 |
| 22 | IC39849 | 0.012 | 0.972 | 0.016 |
| 23 | IC38098 | 0.012 | 0.975 | 0.013 |
| 24 | IC86581 | 0.949 | 0.014 | 0.037 |
| 25 | IC25867 | 0.014 | 0.964 | 0.022 |
| 26 | Eypo | 0.03 | 0.014 | 0.956 |
| 27 | IC74782 | 0.873 | 0.112 | 0.015 |
| 28 | IC74717 | 0.03 | 0.922 | 0.048 |
| 29 | IC67706 | 0.012 | 0.972 | 0.016 |
| 30 | IC52785 | 0.013 | 0.971 | 0.016 |
| 31 | IC67730 | 0.012 | 0.975 | 0.013 |
| 32 | Tsukji | 0.938 | 0.023 | 0.039 |
| 33 | IC115954 | 0.963 | 0.021 | 0.016 |
| 34 | IC12168 | 0.959 | 0.016 | 0.025 |
| 35 | IC74773 | 0.937 | 0.023 | 0.04 |
| 36 | IC36753 | 0.963 | 0.021 | 0.016 |
| 37 | IC134972 | 0.855 | 0.132 | 0.013 |
| 38 | IC8960 | 0.031 | 0.035 | 0.935 |
| 39 | IC27513 | 0.95 | 0.014 | 0.036 |
| 40 | IC330458 | 0.947 | 0.012 | 0.041 |
| 41 | IC35552 | 0.131 | 0.606 | 0.262 |
| 42 | IC45701 | 0.946 | 0.013 | 0.041 |
| 43 | IC19981 | 0.946 | 0.012 | 0.042 |
| 44 | IC66819 | 0.016 | 0.955 | 0.029 |
| 45 | IC209047 | 0.037 | 0.022 | 0.941 |
| 46 | IC54656 | 0.962 | 0.022 | 0.016 |
| 47 | IC145758 | 0.946 | 0.012 | 0.042 |
| 48 | IC17042 | 0.588 | 0.05 | 0.362 |
| 49 | IC35214 | 0.03 | 0.689 | 0.281 |
| 50 | IC25850 | 0.016 | 0.776 | 0.208 |
| 51 | BAM7244 | 0.12 | 0.405 | 0.475 |
| 52 | IC6294 | 0.967 | 0.011 | 0.022 |
| 53 | Jalmagan | 0.027 | 0.015 | 0.958 |
| 54 | IC208092 | 0.039 | 0.02 | 0.94 |
| 55 | IC36704 | 0.949 | 0.014 | 0.036 |
| 56 | BAM8030 | 0.029 | 0.014 | 0.957 |
| 57 | Desitian Raj | 0.538 | 0.054 | 0.408 |
| 58 | IC73121 | 0.029 | 0.015 | 0.957 |
| 59 | BAM7482 | 0.032 | 0.035 | 0.933 |
| 60 | BAM8043 | 0.038 | 0.021 | 0.941 |
| 61 | IC116983 | 0.027 | 0.015 | 0.958 |
| 62 | IC342630 | 0.017 | 0.777 | 0.206 |
| 63 | BAM8032 | 0.954 | 0.01 | 0.036 |
| 64 | IC8177 | 0.039 | 0.021 | 0.941 |
| 65 | CSR30 | 0.957 | 0.018 | 0.025 |
| 66 | IC418922 | 0.031 | 0.035 | 0.934 |
| 67 | BAM7354 | 0.021 | 0.144 | 0.834 |
| 68 | IC8506 | 0.031 | 0.035 | 0.934 |
| 69 | CSR8 | 0.033 | 0.033 | 0.935 |
| 70 | Gujarat5 | 0.027 | 0.015 | 0.958 |
| 71 | IC116989 | 0.019 | 0.053 | 0.928 |
| 72 | IC377621 | 0.343 | 0.011 | 0.645 |
| 73 | Azucena | 0.025 | 0.216 | 0.758 |
| 74 | Camponi sml | 0.037 | 0.094 | 0.87 |
| 75 | IC346909 | 0.023 | 0.012 | 0.965 |
| 76 | BAM8070 | 0.021 | 0.151 | 0.828 |
| 77 | BAM8069 | 0.024 | 0.011 | 0.965 |
| 78 | BAM8068 | 0.027 | 0.015 | 0.959 |
| 79 | Do Dou | 0.03 | 0.034 | 0.937 |
| 80 | IC123723 | 0.944 | 0.013 | 0.043 |
| 81 | IC4434 | 0.949 | 0.014 | 0.036 |
| 82 | IC137492 | 0.945 | 0.012 | 0.043 |
| 83 | BAM8076 | 0.028 | 0.015 | 0.958 |
| 84 | PR116 | 0.134 | 0.014 | 0.852 |
| 85 | BAM8031 | 0.947 | 0.012 | 0.041 |
| 86 | IC38062 | 0.135 | 0.013 | 0.852 |
| 87 | IC61953 | 0.83 | 0.139 | 0.032 |
| 88 | IC253851 | 0.946 | 0.012 | 0.041 |
| 89 | BAM7708 | 0.026 | 0.015 | 0.959 |
